# A Rare Presentation of Left Ventricular Noncompaction Cardiomyopathy Revealed by Acute Decompensated Heart Failure

**DOI:** 10.14740/jmc5284

**Published:** 2026-03-27

**Authors:** Akanksha Sirohi, Sahithi Burra, Saurav Pahal, Sridhar Mangalesh, Laura Marcela Romero Acero, Michele Nanna

**Affiliations:** a Department of Medicine, Luminis Health Anne Arundel Medical Center, Annapolis, MD, USA; b Osmania Medical College, Hyderabad, Telangana, India; c Department of Anesthesia, NYMC Saint Mary’s and Saint Clare’s, Denville, NJ, USA; d Department of Medicine, Jacobi Medical Center, Albert Einstein College of Medicine, Bronx, NY, USA; e Cardiology Division, Cardiac Care and Vascular Medicine, Albert Einstein College of Medicine, Bronx, NY, USA

**Keywords:** Left ventricular noncompaction, Cardiomyopathy, Heart failure, Atrial fibrillation, Echocardiography, Myocardial trabeculation, Systolic dysfunction, Cardiac imaging

## Abstract

Left ventricular noncompaction (LVNC) is an uncommon genetic cardiomyopathy characterized by excessive trabeculations resulting from incomplete myocardial compaction and may remain clinically silent until adulthood. We report the case of a 62-year-old woman with no prior cardiovascular disease who presented with progressive dyspnea, orthopnea, lower extremity edema, and new-onset rapid atrial fibrillation, resulting in acute decompensated heart failure. Initial evaluation revealed elevated blood pressure, irregularly irregular rhythm, pulmonary crackles, and lower limb edema. Transthoracic echocardiography demonstrated a left ventricular ejection fraction of approximately 25%, diffuse hypokinesis, and a distinct two-layered myocardium with prominent apical and lateral trabeculations consistent with LVNC. Coronary angiography ruled out obstructive coronary artery disease, and transesophageal echocardiography confirmed severe systolic dysfunction without atrial thrombus before successful cardioversion. The patient improved with intravenous loop diuretics, initiation of guideline-directed medical therapy, and anticoagulation due to her atrial fibrillation and reduced ejection fraction. This case highlights an uncommon presentation of previously unrecognized LVNC diagnosed in the setting of acute heart failure triggered by arrhythmia, underscoring the importance of echocardiography in differentiating cardiomyopathy phenotypes. Early recognition is essential, as LVNC carries increased risks of arrhythmias, thromboembolic events, and progressive heart failure. Clinicians should maintain a high index of suspicion for LVNC in adults presenting with unexplained systolic dysfunction and excessive trabeculation on imaging, particularly when accompanied by atrial fibrillation.

## Introduction

The cause of left ventricular noncompaction (LVNC), also known as spongy myocardium, is uncertain, although several possible etiological bases have been proposed. There is increasing evidence that supports a genetic basis by identifying mutations in genes that encode sarcomeric, cytoskeletal, and nuclear membrane proteins [1]. The American Heart Association considers it a primary genetic cardiomyopathy [2].

During the development of the heart, prior to the formation of coronary arteries, there are prominent myocardial trabeculations termed sinusoids within the myocardium that serve to increase the surface area for the diffusion of oxygen. Following the development of coronary vasculature, these structures are no longer needed and evolve into a compact myocardium. In some individuals the transformation from spongy myocardium to compact myocardium is incomplete leading to the development of a noncompacted myocardial inner layer with prominent myocardial trabeculations that are continuous with the left ventricular (LV) cavity without communication with the epicardial circulation and a thin epicardial layer [3].

Myocardial trabeculations in LVNC predispose to the formation of ventricular thrombi and arrhythmias, both atrial (5–29%) and ventricular (18–47%). It is associated with high rates of mortality, causing sudden cardiac death, and morbidity in adults, including heart failure, thromboembolic events, and tachyarrhythmias, with heart failure being the most common presentation. Patients often develop progressive symptoms of exertional dyspnea, orthopnea, and lower extremity edema [4]. LVNC is typically diagnosed by echocardiography [5].

This case describes a previously unrecognized LVNC that manifested clinically with new-onset acute decompensated heart failure with reduced left ventricular ejection fraction (LVEF) triggered by sudden onset atrial fibrillation (AF), representing an uncommon presentation that underscores the diagnostic importance of echocardiography in identifying cardiomyopathy phenotypes.

## Case Report

**Figure 1:**
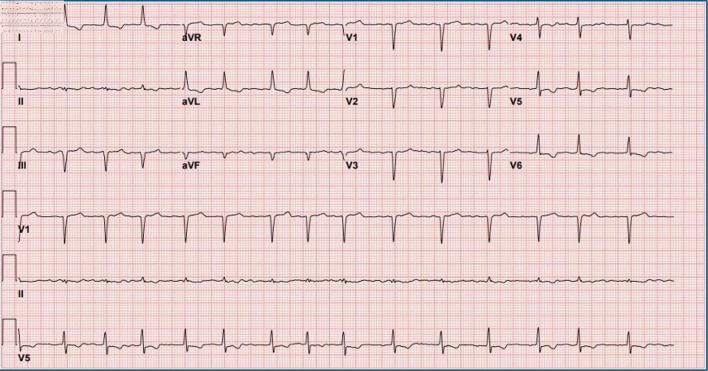
Electrocardiogram on presentation showing atrial fibrillation with rapid ventricular response, inferior Q waves, and probable anteroseptal Q waves.

**Figure 2:**
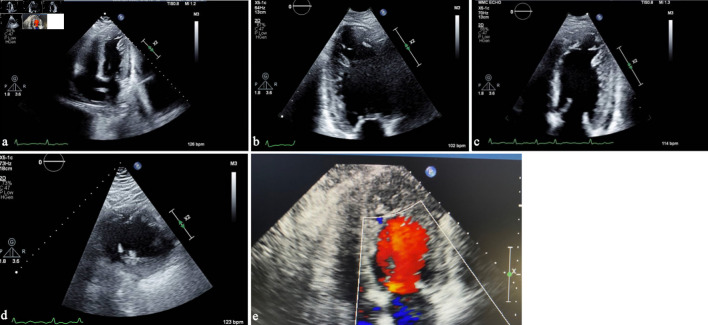
(a) Transthoracic echocardiography, apical four-chamber view, demonstrating prominent trabeculations in the left ventricular apex. (b) Apical two-chamber view showing a two-layered myocardium with deep intertrabecular recesses. (c) Transthoracic echocardiography showing a left ventricular-focused apical four-chamber view illustrating a noncompacted-to-compacted myocardial ratio exceeding 2.0. (d) Parasternal short-axis view illustrating the noncompacted-to-compacted myocardial ratio exceeding 2.0 in systole. (e) Apical four-chamber view focused on the apex showing color-flow Doppler into recesses of apical trabeculations.

A 62-year-old non-smoking female with class II obesity, gastroesophageal reflux disease (GERD), diabetes mellitus complicated by stage 3A chronic kidney disease, and with no prior cardiovascular comorbidities, presented to the emergency room (ER) with dyspnea at rest. The patient had dyspnea on exertion for several months, which worsened acutely over the week prior to admission to the ER, and was associated with shortness of breath (SOB) upon lying down and swelling of both legs. She also reported an episode of chest tightness at rest 1 day before presenting to ER. Upon physical examination, she had an irregularly irregular rhythm, bilateral pitting pedal edema, basilar crackles, and a holosystolic murmur at apex. At presentation, her temperature was 36.9 °C, and her initial blood pressure (BP) was approximately 130–140/80–100. Her electrocardiogram showed AF with rapid ventricular response (Fig. 1) and inferior and anteroseptal Q waves. She was admitted for acute decompensated congestive heart failure (CHF). She had mild leukocytosis on presentation and throughout her hospital course (white blood cell (WBC) 11.9–17.7 × 10^3^/µL). Chest X-ray revealed mild interstitial edema at the left base and excluded pneumonia. Transthoracic two-dimensional (2D) echocardiography showed a LVEF of approximately 25%, severe diffuse LV hypokinesis, normal right ventricular function, and a two-layered endocardium at apex and left lateral portion of the myocardium, suggestive of LVNC cardiomyopathy (Fig. 2).

**Figure 3:**
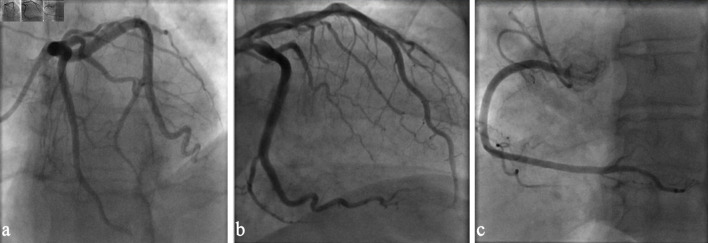
(a) Coronary angiography left anterior oblique view demonstrating patent left main and left anterior descending coronary arteries. (b) Coronary angiography showing unobstructed left circumflex coronary artery. (c) Coronary angiography right anterior oblique view demonstrating patent right coronary artery.

She underwent cardiac catheterization during her hospital stay, which ruled out obstructive coronary artery disease as etiology for the presence of systolic dysfunction (Fig. 3). A transesophageal echocardiogram performed prior to a successful direct-current cardioversion revealed mild left atrial enlargement, excluded the presence of thrombi in left atrium, and confirmed a severely decreased ventricular ejection fraction. A cardiac magnetic resonance imaging (CMR) was not pursued, as the patient declined the study due to associated risks related to her kidney dysfunction. During her hospital course, she was treated with intravenous (IV) loop diuretics and was placed on rivaroxaban (CHA_2_DS_2_-VASc score of 3, female gender, hypertension (HTN), CHF). A beta-blocker, angiotensin receptor-neprilysin inhibitor (ARNI), and sodium-glucose cotransporter 2 (SGLT2) inhibitor were started as part of management for heart failure with reduced ejection fraction. Mineralocorticoid receptor antagonist was not started due to impaired renal function and borderline hyperkalemia. She was discharged clinically stable and symptomatically improved and is being followed as an outpatient for titration of guideline-directed medical therapy for heart failure with reduced ejection fraction.

## Discussion

This case is notable for newly diagnosed LVNC in an elderly woman, a demographic in which this rare cardiomyopathy is infrequently identified. Unlike the more typical presentations in younger, predominantly male patients or those with a known family history, our patient had no prior cardiovascular history and minimal traditional risk factors. The combination of an unusual demographic profile, pseudo-infarct patterns on ECG, and AF unmasking advanced LV dysfunction at the time of diagnosis highlights the wide clinical heterogeneity of LVNC and emphasizes the need for heightened clinical suspicion in patients who fall outside classic risk profiles.

LVNC is a rare form of cardiomyopathy characterized by a thick, spongy myocardial layer with prominent trabeculations and deep intertrabecular recesses. This condition predominantly affects the left ventricle, although right ventricular and biventricular involvement are also documented [6]. Epidemiological studies report the prevalence of LVNC ranging from 0.014% to 0.26% in the general population, with a higher incidence in pediatric populations and individuals with a familial history of cardiomyopathy. Notably, LVNC is observed two to three times more frequently in males than females [7].

The pathophysiology of LVNC is traditionally attributed to an interruption in myocardial compaction during embryogenesis, resulting in the persistence of a noncompacted myocardial layer. Recent evidence, however, suggests that trabecular and compact myocardial layers may develop independently [8]. Genetic mutations, particularly in *MYH7*, *MYBPC3*, and *TTN*, play a significant role in LVNC, affecting proteins integral to sarcomeres, mitochondria, and cellular structure [9]. Moreover, increased preload conditions, such as those occurring in pregnancy or intense athletic training, can lead to LVNC-like myocardial changes [10, 11].

Clinically, LVNC presents a broad spectrum of manifestations. While many patients remain asymptomatic, others develop severe complications, including heart failure, arrhythmias, thromboembolic events, and sudden cardiac death. LVNC is often associated with congenital heart defects and neuromuscular disorders, further complicating its clinical presentation [12].

LVNC is typically identified through noninvasive imaging modalities, most commonly transthoracic echocardiography and CMR. Echocardiography serves as the initial diagnostic tool due to its broad availability. LVNC is diagnosed by marked trabeculations and a noncompacted-to-compacted myocardial ratio exceeding 2.0, although specific echocardiographic criteria and measurement conventions vary across studies, including the different views and whether measurements are taken at end-systole or end-diastole [7]. In our case, the noncompacted-to-compacted ratio clearly surpassed the diagnostic cutoff (Fig. 2). When standard imaging is limited by suboptimal acoustic windows, contrast echocardiography can enhance visualization of the endocardial borders and improve diagnostic reliability [7]. Notably, the diagnostic criteria employed for both echocardiography and CMR remain highly debated, reflecting variability in definitions, measurement techniques, and clinical thresholds.

CMR uses a steady-state free precession sequence to differentiate between the compacted and noncompacted myocardial layers. CMR is more expensive and not widely available and is used to confirm the diagnosis of LVNC when the echocardiographic images cannot provide a definitive diagnosis. It can also detect and quantify myocardial fibrosis, which can predispose to arrhythmia. CMR criteria for diagnosing LVNC require a higher noncompacted-to-compacted myocardium ratio than echocardiography, a ratio greater than 2.3, most commonly seen at end-diastole [3]. It can be seen at end-diastole in either long-axis or short-axis views. CMR was not obtained in this patient per her preference.

Treatment for LVNC is primarily symptomatic and supportive. Management strategies include standard heart failure therapies such as diuretics, angiotensin-converting enzyme (ACE) inhibitors, beta-blockers, and angiotensin receptor blockers. The deep recesses associated with increased trabeculations can be thrombogenic, and anticoagulation is often warranted, especially in the case of prior embolic event, AF, or decreased ejection fraction [13]. For patients at high risk of sudden cardiac death, implantable cardioverter-defibrillators (ICDs) are indicated to manage severe ventricular arrhythmias [3].

The prognosis for LVNC is highly variable. Some patients remain asymptomatic with normal cardiac function, while others may progress to severe heart failure and life-threatening arrhythmias. Mortality rates are notably higher in patients with reduced ejection fraction, frequent arrhythmias, and thromboembolic complications. Prognosis is particularly poor in individuals with associated genetic mutations and neuromuscular disorders [6].

Managing LVNC presents significant challenges due to its variable clinical presentation, genetic heterogeneity, and the absence of standardized diagnostic criteria and treatment guidelines. These challenges underscore the necessity for ongoing research to improve our understanding of the disease, enhance diagnostic accuracy, and develop effective treatment protocols.

## Data Availability

All data supporting the findings of this case report are included within the article. Additional information is available from the corresponding author upon reasonable request.
